# Functional and genetic analysis in type 2 diabetes of Liver X receptor alleles – a cohort study

**DOI:** 10.1186/1471-2350-10-27

**Published:** 2009-03-17

**Authors:** Ingrid Dahlman, Maria Nilsson, Harvest F Gu, Cecile Lecoeur, Suad Efendic, Claes G Östenson, Kerstin Brismar, Jan-Åke Gustafsson, Philippe Froguel, Martine Vaxillaire, Karin Dahlman-Wright, Knut R Steffensen

**Affiliations:** 1 Department of Medicine, Huddinge, Karolinska Institutet, Stockholm, Sweden; 2 Department of Biosciences and Nutrition, Karolinska Institutet, Stockholm, Sweden; 3 Rolf Luft Center for Diabetes Research, Department of Molecular Medicine and Surgery, Karolinska Institutet, Stockholm, Sweden; 4 CNRS UMR 8090 Institute of Biology, Pasteur Institute of Lille and Lille 2 Droit et Santé University, Lille, France; 5 Genomic Medicine, Hammersmith Hospital, Imperial College, London, UK; 6 Center for Nuclear Receptors and Cell Signaling, Department of Cell Biology and Biochemistry, University of Houston, Texas 77 204, USA

## Abstract

**Background:**

Liver X receptor alpha *(LXRA*) and beta (*LXRB*) regulate glucose and lipid homeostasis in model systems but their importance in human physiology is poorly understood. This project aimed to determine whether common genetic variations in *LXRA *and *LXRB *associate with type 2 diabetes (T2D) and quantitative measures of glucose homeostasis, and, if so, reveal the underlying mechanisms.

**Methods:**

Eight common single nucleotide polymorphisms in *LXRA *and *LXRB *were analyzed for association with T2D in one French cohort (N = 988 cases and 941 controls), and for association with quantitative measures reflecting glucose homeostasis in two non-diabetic population-based samples comprising N = 697 and N = 1344 adults. Investigated quantitative phenotypes included fasting plasma glucose, serum insulin, and HOMA_IR _as measure of overall insulin resistance. An oral glucose tolerance test was performed in N = 1344 of adults. The two alleles of the proximal *LXRB *promoter, differing only at the SNP rs17373080, were cloned into reporter vectors and transiently transfected, whereupon allele-specific luciferase activity was measured. rs17373080 overlapped, according to *in silico *analysis, with a binding site for Nuclear factor 1 (NF1). Promoter alleles were tested for interaction with NF1 using direct DNA binding and transactivation assays.

**Results:**

Genotypes at two *LXRB *promoter SNPs, rs35463555 and rs17373080, associated nominally with T2D (P values 0.047 and 0.026). No *LXRA *or *LXRB *SNP associated with quantitative measures reflecting glucose homeostasis. The rs17373080 C allele displayed higher basal transcription activity (P value < 0.05). The DNA-mobility shift assay indicated that oligonucleotides corresponding to either rs17373080 allele bound NF1 transcription factors in whole cell extracts to the same extent. Different NF1 family members showed different capacity to transactivate the *LXRB *gene promoter, but there was no difference between promoter alleles in NF1 induced transactivation activity.

**Conclusion:**

Variations in the *LXRB *gene promoter may be part of the aetiology of T2D. However, the association between *LXRB *rs35463555 and rs17373080, and T2D are preliminary and needs to be investigated in additional larger cohorts. Common genetic variation in *LXRA *is unlikely to affect the risk of developing T2D or quantitative phenotypes related to glucose homeostasis.

## Background

Type 2 diabetes (T2D) is a common cause of morbidity and mortality and is caused by a western sedentary life style with high calory diet in combination with susceptibility genes. Without life style changes, available pharmaceutical treatments of T2D are only partially efficient. Recently, large scale allelic association analyses have begun to identify the genetic factors underlying susceptibility to T2D [1]. This new knowledge may permit unravelling of pathways leading to development of T2D and thereby aid in the development of more efficient prevention and therapy of this disease.

Liver X receptor alpha (*LXRA*, *NR1H3*) and beta (*LXRB*, *NR1H2*) are established sensors of lipid and cholesterol homeostasis [2]. Recently, a large body of literature has indicated a role of LXRs also in glucose metabolism and homeostasis. Several studies have demonstrated that LXR agonists reduce plasma glucose concentrations and increase insulin sensitivity in different models of diabetes and insulin resistance [3,4]. LXRs seem to improve glucose metabolism at different levels. In experimental models, LXR activation inhibits hepatic gluconeogenesis and glucose output [5,6]. Other results support that LXRs regulate peripheral glucose uptake. Activation of LXRs promotes glucose uptake and oxidation in muscle, and expression of the insulin responsive glucose transporter Glucose transporter type 4 (GLUT4) in adipocytes [5,7,8]. LXRs are also implicated in regulation of insulin secretion. Activation of LXRs increases glucose dependent insulin secretion *in vitro *from pancreatic beta-cell line cultures [9]. This effect may be mediated through *LXRB *since *Lxrb*^-/- ^mice have lower basal insulin levels and are glucose intolerant due to impaired glucose induced insulin secretion [10]. Together, these observations point towards a physiological role of LXRs in glucose homeostasis.

We have previously reported that two *LXRB *SNPs associate with obesity in a Swedish cohort [rs35463555 (previously known as LB44732G>A), rs2695121, and marginally, rs17373080 (P value 0.06)] [11]. Furthermore, one *LXRA *SNP (rs11039155) was reported to associate with the metabolic syndrome in two French cohorts [12]. *LXRA *is encoded on human chromosome 11p11.2 and *LXRB *on chromosome 19q13.3.

Here we analyze if eight common SNPs in *LXRA *and *LXRB *associate with T2D in one French case-control cohort, and to phenotypes related to glucose homeostasis in two non-diabetic population-based cohorts comprising adult French and Swedish subjects. We also investigate the impact of one *LXRB *promoter SNP on gene transcription *in vitro*.

## Methods

### Subjects

The present study includes one large French cohort that was used for analysis of association between *LXR *gene SNPs and T2D. In addition, a subset of the non-diabetics in the French cohort, that is D.E.S.I.R., as well as the non-diabetics in the Swedish Stockholm Diabetes Prevention Program (SDPP) cohort, were analyzed for association between *LXR *SNPs and quantitative phenotypes related to glucose homeostasis. The cohorts used for quantitative trait analyses were population based. The study was approved by the ethical committee of the Karolinska University Hospital, the French National Commission for Informatics and Liberty and Hotel Dieu Ethics Committee. Informed written consent was received from all participants.

### French cohort

The French T2D group included 988 unrelated T2D patients (N = 662 males and 326 females, age at examination 61.0 ± 10.2 years, BMI 26.3 ± 2.5 kg/m^2^) recruited at the Endocrinology-Diabetology Department of the Corbeil-Essonnes Hospital [13]. The type 2 diabetes status was defined according to the World Health Organization 1999 criteria [14]. The T2D patients included in the study were selected on a BMI < 30 kg/m^2 ^and all were unrelated. 346 T2D subjects were treated with insulin, 624 were treated with oral anti-diabetic drugs, and 18 subjects had other anti-diabetic treatment. The 941 control subjects were selected on fasting plasma glucose < 6.1 mmol/l and BMI < 27 kg/m^2 ^(N = 380 males and 561 females, age at examination 62.2.0 ± 7.5 years, BMI 23.2 ± 2.0 kg/m^2^) and all were unrelated. Of the 941 controls, 697 were selected from the D.E.S.I.R. general French population [15] and 244 were selected from the French families recruited at the CNRS unit in Lille. All subjects are of French Caucasian origin. We have compared the genotypic distributions within the control group depending on the original selections. No significant difference was observed. For 1021 subjects (case and controls) of this study we could estimate the proportion of European ancestry from a previous genome-wide genotyping [16]. We performed logistic regression taking into account also the gender of the individuals, and observed at least 90% (in 1005 subjects) or 99% (in 835 subjects) of European ancestry (results below not included in the text). Thus, we can potentially exclude a possible stratification in the French study sample. Moreover, these results are supported by several other association studies [16-24]. Information about family history of T2D was available for the French family cohort and for most of the subjects that were included as controls there was no T2D in the ancestry. Family history of T2D was not available from the D.E.S.I.R. participants. The size of the French cohort provided 70% (90%) power to detect an allele with a frequency of 20% among controls and odds ratio (OR) 1.25 (1.35) to develop T2D assuming a threshold P value of 0.05 and dominant mode of inheritance (QUANTO, ) [25].

### Swedish cohort

For the Swedish SDPP study, a short questionnaire was sent to all men born between 1938 and 1957 living in four municipalities in the outskirts of Stockholm and all women born between 1942 and 1961 living in the same municipalities and one additional municipality, asking about country of birth and presence of diabetes in subjects and relatives. Persons were excluded as a result of diabetes and foreign origin. In a second step, subjects with family history of diabetes, together with subjects randomly selected from those without family history of diabetes, matched to the first group by age and municipality were invited to a health examination. Subjects with DNA available were included in this study.

SDPP included 1024 normoglycemic controls with normal glucose tolerance (N = 778 males and 246 females, age at examination 46.7 ± 4.9 years, BMI 25.5 ± 4.4 kg/m^2^) and 320 subjects with impaired glucose tolerance (IGT) (N = 174 males and 146 females, age at examination 48.2 ± 4.5 years, BMI 29.2 ± 5.3 kg/m^2^) [26-29].

The size of the population-based non-diabetic Swedish SDPP and French D.E.S.I.R. cohorts together provided 53% (98%) power to detect an allele with a frequency of 15% and marginal β_G _0.2 (0.4) for HOMA_IR _as measure of insulin resistance, assuming a threshold P value of 0.05 and dominant mode of inheritance (QUANTO, ) [25].

### Clinical evaluation

In the morning a fasting venous blood sample was obtained for determination of fasting levels of insulin and glucose by the routine chemistry laboratory of the hospitals, and for extraction of DNA using standard protocols. An oral glucose tolerance test was performed in the SDPP subjects. Diabetes was defined according to the World Health Organization 1999 criteria: fasting plasma glucose ≥ 7.0 mmol/l and/or 2-h plasma glucose ≥ 11.1 mmol/l, or ongoing treatment with oral antidiabetic agents and/or insulin [14]. IGT was defined as fasting plasma glucose < 7.0 mmol/l and 2-h plasma glucose 7.8–11.0 mmol/l. Insulin resistance index HOMA_IR _(homeostasis model assessment) was calculated as fasting serum insulin (μU/ml) × fasting plasma glucose (mmol/l)/22.5 [30]. The diagnosis of T2D as opposed to type 1 diabetes was based on clinical records and judgment by the investigating physicians.

### SNP selection and genotyping

**Figure 1:**
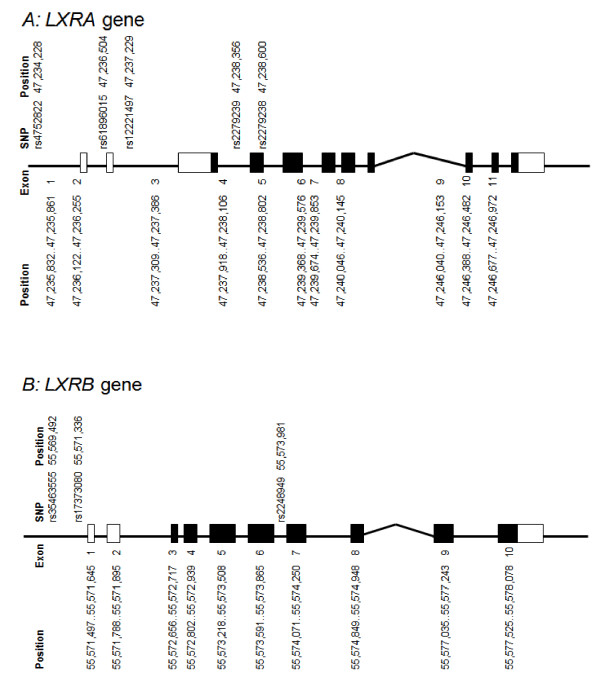
**Schematic figure of *LXRA *and *LXRB *genes with position of genotyped SNPs according to NCBI as indicated**.

We genotyped altogether five SNPs in the *LXRA *gene (Figure 1A). Three of the genotyped *LXRA *SNPs cover the common variation in this gene in a French cohort according to Legry et al [12]. According to Legry et al a, *LXRA *comprises three common haplotypes, which are tagged by SNPs genotyped in the present study. We were unable to design a multiplex assay including the SNP rs11039155, which in French cohorts has been associated with the metabolic syndrome. However, we genotyped instead rs2279238 that is in complete linkage disequilibrium (LD) with rs11039155 [12]. Since the SNPs genotyped by Legry did not fully cover the two untranslated 5' exons of *LXRA *we added two more SNPs in this region from our previous study of *LXRA *in obesity, that is rs4752822 and rs61896015 (the latter previously labelled LA9462C>A) [11]. For *LXRB*, we genotyped three SNPs that covered 57% of alleles as r^2 ^≥ 0.80 according to our own sequencing and genotyping of the gene in a Swedish cohort (Figure 1B) [11]. The reason for the relatively low coverage was that we were unable to design a high throughput genotyping assay for two variations; a repeat and a SNP located in this repeat. The three SNPs cover the three common *LXRB *gene haplotypes according to our own previous genotyping of this gene in a Swedish cohort [11].

Both samples were genotyped using matrix-assisted laser desorption/ionization time-of-flight (MALDI-TOF) mass spectrometry [31]. Primers are provided on request. Due to initial low call rate, rs2248949 was re-genotyped by Taqman in a subset of the Swedish cohort (C_1993248_20, Applied Biosystems, Foster City, CA, USA). The final genotype call rate was ≥ 93% for all SNPs. The accuracy was 99.99% according to duplicate analysis of, on average, 2% of the total genotypes. Hardy-Weinberg equilibrium (HWE) calculations were performed in Haploview to ensure that each marker was within population equilibrium [32].

### Cloning of the *LXRB *promoter into reporter vectors and *in vitro *mutagenesis

The -244/+1163 sequence of the human *LXRB *gene was cloned into the pGL3 basic luciferase reporter vector (Promega) using the *Kpn *I and *Mlu *I sites applying primers 5'-ATCAGGTACCGGCCGCAGGCTCAGAGAAGCGCATGAATGAGCTAA-3' and 5'-ATCACTCGAGGGTGGGGTCACGGAGCAGCCTGTAGAATACAGGGGATTGAGAG-3' with the restriction enzyme sites underlined. The G allele (bold) of rs17373080 was generated using the following primer (5'-TAAAGCCA***G***AAAGCGCGGGGCTGGAGGTTT-3' using the QuikChange^® ^XL Site-Directed Mutagenesis Kit (Stratagene). DNA sequencing confirmed the identity of all clones.

### Transient transfections

The mouse pancreatic beta-cell line MIN6 was maintained in Dulbecco's modified Eagle's medium (DMEM, 4.5 g/L glucose, 41965-039), the human hepatoma cell line HuH7 was grown in DMEM with 0.11 G/L NaPyr (41966-029), and African green monkey kidney cells Cos7 in DMEM with 1 g/L glucose (31885-023). All media were from GIBCO-BRL. The cell lines were supplemented with fetal bovine serum (Cos7, HuH7: 10%, MIN6: 15%), 2 mM L-glutamine (only MIN6), and penicillin/streptomycin at a final concentration of 100 U/ml and 100 μg/ml, respectively. Additionally, 50 μM β-mercaptoethanol was added to the MIN6 cells. Cells were grown under 5% CO_2 _at 37°C. Approximately 4 × 10^4 ^cells per well were plated in 24 well plates and transiently transfected using Lipofectamine 2000 (Invitrogen, Carlsbad, CA) according to the manufacturer's protocol. Each well received 50 ng of reporter vector and 125 ng of expression vector, respectively. Vehicle vector was added to ensure equal amounts of DNA in each transfection. Cells were transfected for 24 h and thereafter lysed in 25 mM TAE, 1 mM EDTA, 10% glycerol, 1% Triton X-100, and 2 mM DTT. Luciferase activities were measured using a Luciferase Assay Kit (BioThema, Umeå, Sweden) in a luminometer (Luminoscan Ascent, Thermo electron Corporation, Waltham, MA). The NF1 transcription factors were cloned into the pCH-expression vectors and were a kind gift of Dr. Gronostajski, Cleveland Clinic Foundation Research Institute.

### Whole cell extracts

MIN6 cells were grown to confluence in 100 mm dishes, washed with PBS and incubated in TEN buffer (40 mM Tris-HCl, 1 mM EDTA, 150 mM NaCl) for 4 min. Cells were then removed with a cell scraper and pelleted by centrifugation at 3,500 rpm for 2 min at 4°C. Cell pellets were freeze dried on dry ice and resuspended in 50 μl ice-cold buffer C (10 mM Hepes-KOH pH 7.9, 0.4 M NaCl, 0.1 mM EDTA, 5% glycerol, 1 mM DTT, 0.5 mM PMSF). After another round of freeze drying, cell debris were removed by centrifugation for 5 min at 13,000 rpm at 4°C. The supernatant corresponds to MIN6 whole cell extracts.

### Electro mobility shift assay (EMSA)

The oligo including a consensus site for nuclear factor 1 (NF1) is 5'-GATCTTATTTTGGATTGAAGCCAATATGAG-3'. The rs17373080 C allele oligo is 5'-GCTAAAGCCA***C***AAAGCGCGGGG-3' with the C allele in bold. The rs17373080 G allele oligo is 5'-GCTAAAGCCA***G***AAAGCGCGGGG-3' with G allele in bold. 5.0 μg of the respective forward and reverse oligos were annealed in 20 mM Tris-HCl pH 7.8; 2 mM MgCl_2_; 50 mM NaCl by heating to 95°C for 5 min and slow cooling by 1.5°C/min for 47 cycles. Oligonucleotide probes were labeled by mixing 0.2 μg annealed oligo with 250 μM non-radioactive dATP, dGTP, dTTP respectively, 1× Klenow buffer, 20 μCi ^32^P-labeled dCTP (Amersham Pharmacia) and 1 Unit Klenow polymerase. Samples were incubated for 20 min at room temperature. Reactions were stopped by adding 0.5 M EDTA. Probes were purified using G-25 Nick Columns (Amersham Pharmacia) and the efficiency of labeling determined using the 1214 Rackbeta liquid scintillation counter (LKB Wallac). For binding reactions, 5 μl of MIN6 whole cell extracts were incubated with 4 × 10^4 ^cpm of radiolabeled oligonucleotide in binding buffer (4 μg BSA, 2 μg poly dI-dC, 12 mM HEPES (pH 7.9), 12% glycerol, 0.12 mM EDTA, 0.9 mM MgCl_2_, 0.6 mM dithiothreitol, 0.6 mM phenylmethylsulfonyl fluoride). Binding reactions were incubated for 20 min at RT, electrophoresed at 150 V for 3 h in 6% polyacrylamide gels at 4°C, dried and finally analyzed by autoradiography. In competition assays, the unlabeled competitor was added prior to the nuclear extracts.

### Statistical analyses

The Finetti software and Pearson's Chi^2 ^(d.f. = 1) were used to compare allele and genotype frequencies between cases and controls and to calculate odds ratios (OR) [33]. The genetic models analyzed by the Finetti software were defined as homozygous/recessive: (Case_11*Control_22)/(Case_22*Control_11) and allele positivity/dominant: ((Case_12+Case_11)*Control_22)/(Case_22*(Control_12+Control_11)). To take into account the impact of gender on susceptibility to T2D, we used logistic regression with genotype and sex as independent variables. Haploview was used to calculate LD between SNPs (D' and r^2^), estimate haplotypes, and to test for association between haplotypes and T2D [32]. The program Thesias was used to test two-by-two sliding window SNPs haplotypes [34]. This program determines both the haplotype frequency and its effect on the phenotype through an estimation-maximisation algorithm. In this analysis, we also considered gender as a covariate.

ANCOVA with SNP genotypes and gender as main effects and age, and when appropriate BMI, as simple regressor was used to analyze differences in HOMA_IR_, fasting plasma glucose, serum insulin, and BMI between *LXR *genotypes in the non-diabetic population-based cohorts, SDPP and normoglycemic non-obese samples from the D.E.S.I.R. respectively. The analyses were repeated in joint analyses of both cohorts including cohorts as an additional main effect. 2-h plasma glucose was available and analyzed as above in the Swedish cohort only. Quantitative phenotypes were normalized by natural logarithm transformation before analysis to become normally distributed. In the transient transfections functional studies the student's t-test was applied.

## Results

### Analysis of association between *LXR *gene SNPs and T2D

**Figure 2:**
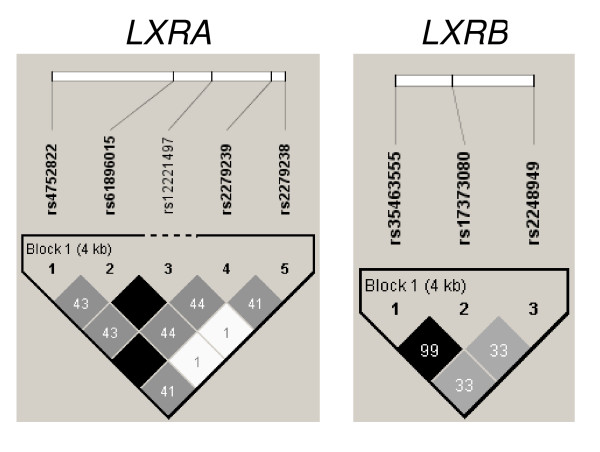
**Linkage disequilibrium between *LXRA *and *LXRB *SNPs as indicated**. Shown are r^2 ^values. D' was 1.00 between all SNPs within each gene.

We first analyzed *LXRs *SNPs for association with T2D in the French case-control cohort. Altogether we genotyped five *LXRA *SNPs and three *LXRB *SNPs. All SNPs had a call rate of ≥ 93% and were in HWE in the French cohort (Table 1). We analyzed *LXR *SNPs for association with T2D under different genetic models. No SNP allele associated to T2D (Table 1). However, genotype at the *LXRB *promoter SNPs rs35463555 and rs17373080 associated nominally to T2D assuming a dominant T2D-predisposing common allele, P value 0.047 and 0.026 respectively (Table 2). This was due to an increased risk of T2D among heterozygous and protection against T2D among subjects homozygous for the rare allele (Table 1). None of analyzed SNPs showed significant association with T2D when taking into account the gender effect. SNPs within *LXRA *and *LXRB *were in strong LD and together built one haploblock for the respective genes (Figure 2). No *LXRA *and no *LXRB *haplotype associated with T2D (Table 3). Furthermore, no two-by-two sliding window SNPs haplotypes associated with T2D (results not shown).

**Table 1:** Analysis of association between *LXR *alleles and T2D in French cases vs control

**SNP**									**Call**	**HWE**	
**Name**	**Position**	**Allele^a^**		**#11^b^**	**#12**	**#22**	**%1^c^**	**%2**	**rate %**	**P value^d^**	**O.R. ± C.I**.^e^

***LXRA***											

rs4752822	5'	T>C	ND^g^	522	332	53	76	24	96	0.93	1.04 [0.89–1.21]
			
			T2D^g^	537	360	57	75	25			

rs61896015	intron 2	C>A	ND	711	180	16	88	12	97	0.79	1.13 [0.93–1.37]
			
			T2D	729	221	15	87	13			

rs12221497	intron 2	G>A	ND	718	180	17	88	12	97	0.69	1.09 [0.90–1.33]
			
			T2D	727	216	13	87	13			

rs2279239	intron 4	T>C	ND	518	328	52	76	24	97	0.94	1.03 [0.89–1.20]
			
			T2D	549	355	60	75	25			

rs2279238	exon 5	C>T	ND	715	195	12	88	12	98	0.62	0.99 [0.81–1.2]
			
			T2D	754	192	17	88	12			

***LXRB***											

rs35463555	promoter	G>A	ND	417	388	106	67	33	96	0.74	0.94 [0.82–1.08]
			
	(-2046)		T2D	433	432	84	68	32			

rs17373080	promoter	C>G	ND	411	386	104	67	33	96	0.42	0.94 [0.82–1.08]
			
	(-202)		T2D	430	438	80	68	32			

rs2248949	intron 6	C>T	ND	310	432	139	60	40	93	0.05	1.02 [0.90–1.17]
			
			T2D	303	476	136	59	41			

a) Common allele is written first. b) #11 = number of subjects homozygous for common allele, #12 = number of heterozygous subjects, #22 = number of subjects homozygous for rare allele. c) Major allele frequency. d) HWE P values were calculated in HAPLOVIEW [32]. e) O.R. ± C.I. = odds ratio ± confidence intervals. ORs were calculated as (number allele 2 among cases * number allele 1 among controls)/(number allele 1 among cases * number allele 2 among controls). f) Pearson's Chi^2 ^(d.f. = 1) was used to compare allele frequencies between T2D-cases and controls. g) ND = non-diabetic controls, T2D = type 2 diabete

**Table 2:** Association of *LXRB *promoter SNP genotypes with T2D in the French cohort

	Recessive		Dominant	
	**O.R.+C.I**. [22]<->[11]	P value	**O.R.+C.I**. [22]<->[11+12]	P value

rs35463555	1.310 [0.955–1.798]	0.09	1.356 [1.003–1.834]	0.047

rs17373080	1.360 [0.986–1.875]	0.06	1.416 [1.042–1.924]	0.026

1 = common allele, 2 = rare allele

OR: homozygous/recessive: (Case_11*Control_22)/(Case_22*Control_11), allele positivity/dominant: ((Case_12+Case_11)*Control_22)/(Case_22*(Control_12+Control_11))

**Table 3:** Analysis of *LXR *haplotypes in T2D in the French cohort

**Block**	**Specific haplotype (%)**		**nominal**
	**Case**	**Controls**	**P Value**

*LXRA*: rs4752822- rs61896015- rs12221497- rs2279239- rs2279238			

TCGTC	75.1	75.9	0.56

CAACC	13.1	12.0	0.32

CCGCT	11.8	12.1	0.80

*LXRB*: rs35463555- rs17373080- rs2248949			

GCT	41.0	40.6	0.81

AGC	31.7	33.0	0.41

GCC	27.2	26.4	0.55

Haplotype frequencies in cases versus controls are compared by Chi^2 ^test. Counts for association tests are obtained by summing the fractional likelihoods of each individual for each haplotype.

### Analysis of association between *LXR *SNPs and quantitative phenotypes reflecting glucose homeostasis

We next analyzed *LXR *gene SNPs for impact on quantitative phenotypes related to glucose homeostasis in non-diabetic subjects. For this purpose, we analyzed the normoglycemic non-obese population-based samples selected from the French D.E.S.I.R. cohort and the Swedish SDPP cohort. Phenotypes of these cohorts are shown in Table 4. In the Swedish cohort an oral glucose tolerance test was performed and the analyzed cohort included 320 subjects with IGT. Genotyping result for the D.E.S.I.R. samples is included in the controls in Table 1. Genotype results for SDPP are shown in Table 5. All SNPs had call rates ≥ 96%. All SNPs were in HWE. Allele frequencies for *LXRB *SNPs were almost identical in the Swedish and French cohorts whereas allele frequencies for *LXRA *SNPs differed 4–10% between the populations. *LXR *SNPs had no impact on fasting plasma glucose or serum insulin, insulin resistance measured as HOMA_IR _or BMI in either the D.E.S.I.R. or the SDPP cohort, nor in the joint analysis of both cohorts (results not shown). Nor did *LXR *SNPs associate to the response to an OGTT in SDPP (results not shown).

**Table 4:** Phenotypic distribution of cohorts

French	T2D cases					
	all		females		males	

number	988		326		662	
Age (year)	61.0 ± 10.2		62.8 ± 10.7		60.1 ± 9.9	
BMI (kg/m^2^)	26.3 ± 2.5		26.0 ± 2.8		26.4 ± 2.3	
fP-Glucose (mmol/l)	NA		NA		NA	
fS-Insulin (pmol/l)	NA		NA		NA	
HOMA_IR_	NA		NA		NA	

French	Normoglycemic controls					

	D.E.S.I.R.			From the CNRS unit in Lille		

	all	females	males	all	females	males

number	697	419	278	244	142	102
Age (year)	53.4 ± 5.6	53.2 ± 5.7	53.8 ± 5.6	60.9 ± 10.5	61.2 ± 10.9	60.6 ± 10.0
BMI (kg/m^2^)	23.2 ± 1.8	22.8 ± 1.7	23.9 ± 1.7	23.0 ± 2.8	22.4 ± 3.1	23.7 ± 2.0
fP-Glucose (mmol/l)	5.1 ± 0.4	5.0 ± 0.4	5.2 ± 0.3	5.0 ± 0.4	5.0 ± 0.4	5.2 ± 0.4
fS-Insulin (pmol/l)	5.1 ± 3.7	4.9 ± 2.3	5.3 ± 5.2	7.4 ± 5.2	7.1 ± 4.6	7.7 ± 5.9
HOMA_IR_	1.3 ± 0.6	1.3 ± 0.6	1.4 ± 0.7	1.7 ± 1.2	1.6 ± 1.0	1.8 ± 1.4

Swedish	SDPP					

	Normoglycemic controls			Impaired glucose tolerance		

number	1024	246	778	320	146	174
Age (year)	46.7 ± 4.9	47.0 ± 4.6	46.6 ± 5.0	48.2 ± 4.5	48.6 ± 4.4	48.0 ± 4.6
BMI (kg/m^2^)	25.5 ± 4.4	22.6 ± 1.7	26.5 ± 4.5	29.2 ± 5.3	29.2 ± 6.0	29.2 ± 4.6
fP-Glucose (mmol/l)	4.6 ± 0.5	4.5 ± 0.4	4.7 ± 0.6	5.3 ± 0.7	5.2 ± 0.6	5.5 ± 0.7
fS-Insulin (pmol/l)	18.0 ± 10.2	9.2 ± 3.6	20.7 ± 10.0	22.0 ± 11.8	15.6 ± 7.6	27.1 ± 12.1
HOMA_IR_	3.7 ± 2.3	1.8 ± 0.8	4.3 ± 2.3	5.2 ± 3.2	3.5 ± 2.1	6.6 ± 3.2
2 h P-Glucose (mmol/l)	4.5 ± 1.2	4.1 ± 1.0	4.7 ± 1.3	8.7 ± 1.0	8.8 ± 0.8	8.7 ± 1.1

Values are mean ± SD. NA = not available

**Table 5:** *LXR *genotypes in the Swedish population based SDPP cohort

**SNP**	**Allele^a^**	**#11^b^**	**#12**	**#22**	**%11**	**%12**	**%22**	**%1^c^**	**%2**	**call rate %**	**HWE** **P value^d^**
*LXRA*											

rs4752822	T>C	570	605	157	43	45	12	66	34	99	0.91

rs61896015	C>A	886	377	47	68	29	4	82	18	97	0.42

rs12221497	G>A	902	370	47	68	28	4	82	18	98	0.27

rs2279239	T>C	559	590	151	43	45	12	66	34	97	0.86

rs2279238	C>T	923	360	35	70	27	3	84	16	98	1.00

*LXRB*											

rs35463555	G>A	612	576	140	46	43	11	68	32	99	0.86

rs17373080	C>G	613	578	137	46	43	11	68	32	99	1.00

rs2248949	C>T	426	607	267	33	47	20	56	44	97	0.07

a) Common allele is written first. b) #11 = number of subjects homozygous for common allele, #12 = number of heterozygous subjects, #22 = number of subjects homozygous for rare allele. c) Major allele frequency. d) HWE P values were calculated in HAPLOVIEW [32].

### Functional analysis of the *LXRB *rs17373080 SNP

#### The C allele of rs17373080 associates with higher transcription activity in vitro

**Figure 3:**
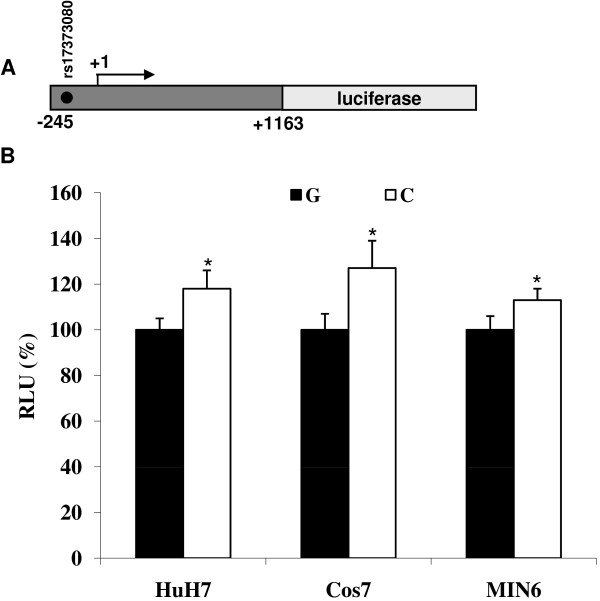
**A. Schematic view of the reporter gene construct driven by the endogenous -244/+1163 LXRB gene region**. The black dot indicates rs17373080. B. Basal activities of the -244/+1163 rs17373080 G allele and C allele reporter constructs. Using *in vitro *mutagenesis with the G allele construct as a template a new C allele construct was generated. The results presented are mean differences of more than 20 independent transfection experiments. The activities of the G allele in each cell line analyzed were set to 100% ± SEM. * P < 0.05 by Student's *t *test.

We have previously determined the transcription start site of the human *LXRB *gene promoter [35]. The *LXRB *SNP rs17373080 is located in the proximal promoter close to the transcription start site and could potentially have an impact on the transcription potential of the *LXRB *gene promoter. Hence we performed functional studies investigating this SNP. The -244/+1163 gene region including the rs17373080 with either the C or the G allele was PCR cloned in front of the luciferase gene reporter (Figure 3A). Otherwise the promoters were identical. The activities of the two different alleles were analyzed in transient transfection assays in three different cell lines. There was a significantly higher basal activity for the C allele in all three cell lines investigated (Figure 3B) indicating that the C allele is able to maintain a higher basal transcription of the *LXRB *gene.

#### A functional NF1 binding site overlaps rs17373080

**Figure 4:**
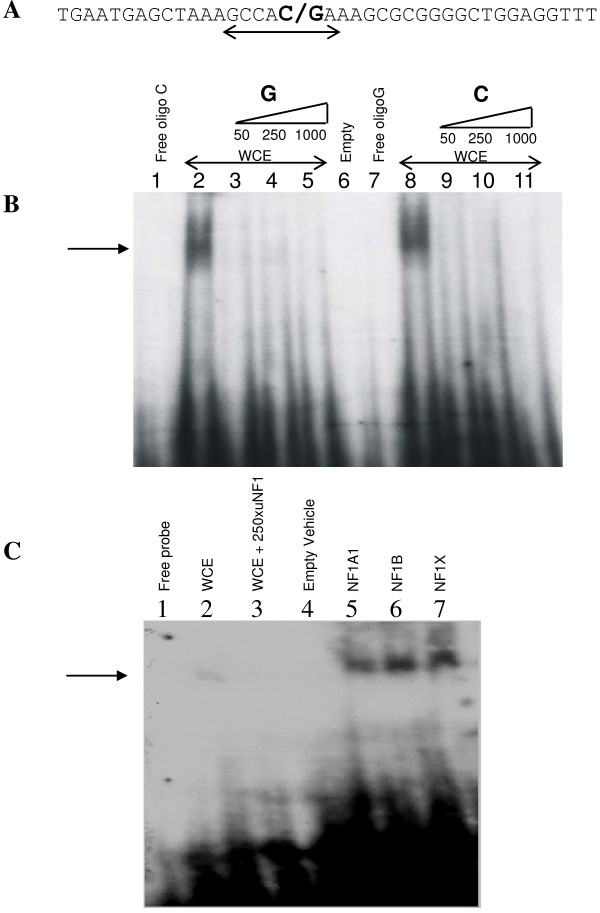
**A. A putative NF1 binding site in the promoter region of the LXRB gene as suggested by TESS (underlined) **[36]. The rs17373080 SNP is indicated in bold. **B**. EMSA with MIN6 whole cell extracts (WCE) and labelled oligonucleotides with the rs17373080 C and G variants, respectively. Lane 1: Free oligo C, 2: Oligo C + MIN6 WCE, 3–5: Oligo C + MIN6 WCE + increasing amounts of unlabeled NF1 consensus oligonucleotide, 6: Empty 7: Free oligo G, 8: Oligo G + MIN6 WCE, 9–11: Oligo G + MIN6 WCE + increasing amounts of unlabeled NF1 consensus oligonucleotide. **C**. EMSA with rs17373080 SNP oligo C and MIN6 whole cell extract or in vitro translated (IVT) NF1. Lane 1: Free oligo C, 2: Oligo C + MIN6 whole cell extract, 3: Oligo C + MIN6 whole cell extract + 250× unlabeled NF1 consensus oligonucleotide, 4: Oligo C + Empty IVT expression vector (vehicle) and 5–7: Oligo C + IVT NF1A1, NF1B and NF1X, respectively. The arrows indicate the positions of the NF1 interactions.

**Figure 5:**
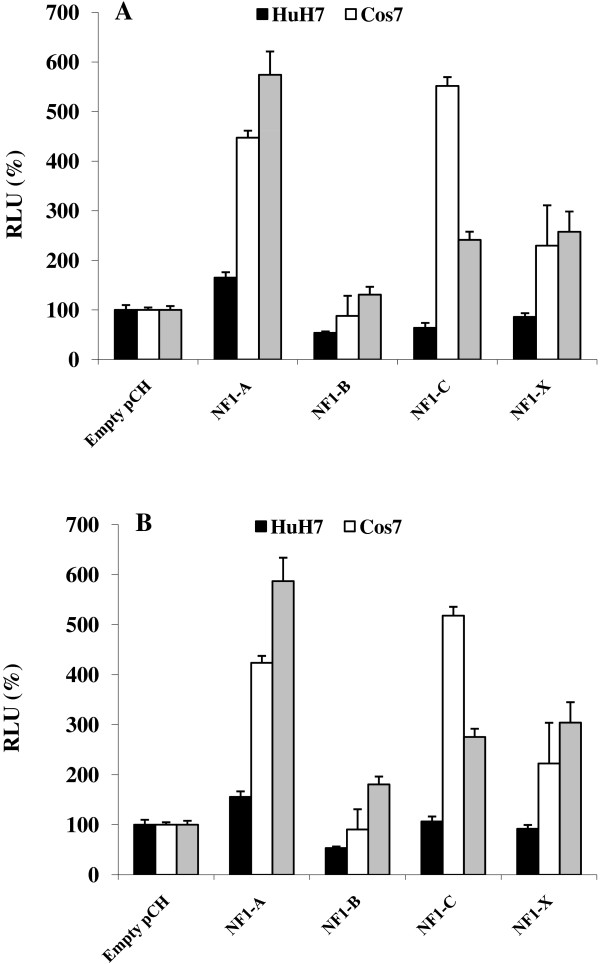
**Transient co-transfections with the -244/+1163 C (A) or G (B) reporter constructs and members of the NF1 family of transcription factors in three different cell lines as indicated**. Each transient transfection was made in triplicates and the results presented are mean differences of three independent experiments. C or G alleles with co-transfection of empty pCH-vector were set as controls. Both C and G allele controls are set to 100% and the effect of NF1 expression compared to either the C-Control **(A) **or G-Control **(B)**. * indicated significant difference (P < 0.05) ± SEM.

A theoretical search for transcription factor binding sites using the transcription element search system (TESS) suggested a binding site for the NF1 transcription family members overlapping the rs17373080 position (Figure 4A) [36]. The different alleles were tested for interaction with NF1 using direct DNA binding and transactivation assays. The DNA-mobility shift assay indicated equal binding of components in whole cell extracts to oligonucleotides corresponding to the C and G alleles, respectively (Figure 4B, lanes 2 and 8) and the DNA-protein interaction was competed away by increasing amounts of an unlabelled NF1 consensus binding site (lanes 3–5 and 9–11). NF1A1, NF1B and NF1X produced by *in vitro *transcription/translation gave protein-DNA complexes of the same size as that produced by whole cell extracts (results not shown). Different NF1 family members (NF1A1, NF1B, NF1C and NF1X) showed different transactivation capacity on the *LXRB *gene promoter (Figure 5). However, no difference in induced transactivation activity of the NF1s was observed between the G (Figure 5A) and C (Figure 5B) promoter alleles.

## Discussion

We have analyzed common genetic variation in *LXRA *and *LXRB *for association with T2D in one French cohort and for association with quantitative measures of glucose homeostasis in two non-diabetic population-based samples from France and Sweden. Genotype at the *LXRB *promoter SNPs rs17373080 and rs35463555 nominally associates to T2D. rs17373080, which regulates transcription *in vitro*, may directly affect the risk of developing T2D whereas the association between T2D and rs35463555, which is located in a repeat, could be due to strong LD with 17373080. No *LXRA *SNPs associate to the investigated phenotypes.

We have previously reported that *LXRB *SNPs associate with obesity in a Swedish cohort [rs35463555 (previously known as LB44732G>A), rs2695121, and marginally, rs17373080 (P value 0.06)] [11]. The genotype at rs35463555 and rs17373080 predisposing to obesity in the previous study was found to also associate to T2D in this study. The association between rs35463555, rs17373080 and T2D reported here would become non-significant following Bonferroni-correction for analysis of several *LXR *SNPs and phenotypes. However, since our results are confirmatory in nature, that is the rs35463555 and, marginally, rs17373080 SNPs have previously been associated with another metabolic phenotype, namely obesity, we think it is too stringent to perform Bonferroni correction. Similarly, since analysed phenotypes are dependent, Bonferroni correction may be too stringent. On the other hand, it is important to note that observed association P values for rs35463555 and rs17373080 (0.047 and 0.026) are weak and would become non-significant following adjustment for just two independent analyses. We did not observe any association between *LXRB *SNPs and BMI in this study. This may be due to phenotypic differences between cohorts. The population-based cohorts D.E.S.I.R. and SDPP that we analyzed here contain markedly less obese people than the previous Swedish study. Together, previous and present results support that *LXRB *promoter genotype regulates susceptibility to adiposity as well as T2D. However, replications of these associations in additional large cohorts are necessary before establishing an association between *LXRB *genotype, obesity and T2D. Published genome wide association studies of T2D have not reported association with *LXR *genes [16-24,37-39]. However, it is uncertain to what extent these studies cover the rather small *LXRA *and *LXRB *genes since the full datasets are unavailable. In the French cohort, gender distribution was the opposite in T2D cases versus controls. The main concern with different gender distribution in cases and controls is reduced power to identify T2D genes due to gender impact on T2D susceptibility and potential gene-gender interaction.

An *LXRB *promoter SNP could contribute to development of T2D by altering mRNA levels of *LXRB *in one or more of the organs regulating glucose homeostasis; *LXRB *is expressed in pancreas, liver, muscle, as well as adipose tissue [40]. It has recently been reported that LXRB is the main LXR paralogue in pancreatic beta-cells, that activated LXR induces insulin secretion from beta-cells and that mice lacking the *Lxrb *gene have less glucose stimulated insulin secretion [9]. The T2D-associated *LXRB *promoter SNP rs35463555 is located in a large repeat region of ~2500 base pairs and is therefore difficult to clone and study functionally. An *in silico *search using TESS revealed no binding sites for human transcription regulators covering rs35463555 [36]. Therefore, we did not perform any functional studies of this SNP. As for the proximal *LXRB *promoter SNP rs17373080, according to our results the GG genotype protected against T2D whereas the G allele showed reduced reporter gene activity, which suggests that the G allele is associated with lower *LXRB *mRNA levels. Thus, these results are contrary to what one would expect based on the results in the *Lxrb *knock-out mice. However, rs17373080 may regulate overall glucose homeostasis through other mechanisms and in other organs in humans.

We observed that the rs17373080 SNP overlapped an NF1 site. The NF1 site was functional in recruiting NF1. However, we did not observe any differences between the G and C alleles in DNA-binding and transactivation of a reporter gene by NF1 family members. It cannot be excluded that rs17373080 overlaps additional transcription factor binding sites and that the binding and/or activity of these sites is affected by the various rs17373080 alleles. It might also be that the differences between the C and G alleles with regard to transcription factor-induced transcription activity are too subtle to be detected with existing methods. However, our results indicate that the transcriptional activity of NF1 could be tissue or cell specific as similar results were observed in Cos7 and MIN6 cells, but not in HuH7 cells. Speculatively, these results could suggest that the relevance of the SNP rs17373080 in developing T2D could be tissue specific and further studies should be focusing on the role of LXRB in these cell types where NF1 was shown to affect the transcription rate of the *LXRB *gene.

There was no association between SNPs covering the common variation in *LXRA *and susceptibility to T2D or levels of plasma glucose, serum insulin, and insulin resistance measured as HOMA_IR_. According to power calculations our cohorts were large enough to detect also a modest impact of *LXRA *on these phenotypes and it is therefore unlikely that this gene directly regulates glucose homeostasis. We did not have access to blood lipids in the SDPP cohort. This prohibited us from determining which subjects fulfilled the criteria for the metabolic syndrome and from analysing *LXR *genotypes for influence on this phenotype. We hypothesize, based on previously reported association of *LXRA *alleles with the metabolic syndrome and HDL levels, together with the lack of association with T2D and measures of glucose homeostasis reported here, that the impact of *LXRA *on susceptibility to the metabolic syndrome is mediated via an impact on lipid turnover rather than glucose homeostasis [12].

## Conclusion

The common *LXRB *promoter SNPs rs35463555 and rs17373080 may regulate susceptibility to T2D, whereas common genetic variation in *LXRA *is unlikely to affect the risk of developing T2D or quantitative phenotypes related to glucose homeostasis. The association between *LXRB *rs35463555 and rs17373080 and T2D needs to be investigated in additional large cohorts.

## Competing interests

Jan-Åke Gustafsson is consultant, shareholder and research grant receiver of KaroBio AB. The other authors declare that they have no competing interests.

## Authors' contributions

ID analyzed the data and wrote the manuscript. MN designed the study and performed the *in vitro *studies. HFG, MV and CL participated in the design of the study, data analysis, and in the writing of the manuscript. CCP, SE, CGO, KB, GC and PF ascertained the patient cohorts. JÅG helped to draft the manuscript. KDW and KRS designed the study and helped to draft the manuscript. All authors have read and approved the final manuscript.

## Pre-publication history

The pre-publication history for this paper can be accessed here:


